# Diagnostic Accuracy of 2D-Shear Wave Elastography for Liver Fibrosis Severity: A Meta-Analysis

**DOI:** 10.1371/journal.pone.0157219

**Published:** 2016-06-14

**Authors:** Tian’an Jiang, Guo Tian, Qiyu Zhao, Dexing Kong, Chao Cheng, Liyun Zhong, Lanjuan Li

**Affiliations:** 1 Department of Ultrasonography, First Affiliated Hospital, Zhejiang University School of Medicine, Hangzhou 310003, China; 2 State Key Laboratory for Diagnosis and Treatment of Infectious Diseases, Collaborative Innovation Center for Diagnosis and Treatment of Infectious Diseases, The First Affiliated Hospital, Zhejiang University School of Medicine, Hangzhou 310003, China; 3 Department of Hepatobiliary Pancreatic Surgery, First Affiliated Hospital, Zhejiang University School of Medicine, Hangzhou 310003, China; 4 Department of Mathematics, Zhejiang University, Hangzhou 310027, China; University Medical Center of Princeton/Rutgers Robert Wood Johnson Medical School, UNITED STATES

## Abstract

**Purpose:**

To evaluate the accuracy of shear wave elastography (SWE) in the quantitative diagnosis of liver fibrosis severity.

**Methods:**

The published literatures were systematically retrieved from PubMed, Embase, Web of science and Scopus up to May 13^th^, 2016. Included studies reported the pooled sensitivity, specificity, positive and negative predictive values, as well as the diagnostic odds ratio of SWE in populations with liver fibrosis. A bivariate mixed-effects regression model was used, which was estimated by the *I*^*2*^ statistics. The quality of articles was evaluated by quality assessment of diagnostic accuracy studies (QUADAS).

**Results:**

Thirteen articles including 2303 patients were qualified for the study. The pooled sensitivity and specificity of SWE for the diagnosis of liver fibrosis are as follows: **≥**F1 0.76 (*p*<0.001, 95% CI, 0.71–0.81, *I*^*2*^ = 75.33%), 0.92 (*p*<0.001, 95% CI, 0.80–0.97, *I*^*2*^ = 79.36%); **≥**F2 0.84 (*p* = 0.35, 95% CI, 0.81–0.86, *I*^*2*^ = 9.55%), 0.83 (*p*<0.001, 95% CI, 0.77–0.88, *I*^*2*^ = 86.56%); **≥**F3 0.89 (*p* = 0.56, 95% CI, 0.86–0.92, *I*^*2*^ = 0%), 0.86 (*p*<0.001, 95% CI, 0.82–0.90, *I*^*2*^ = 75.73%); F4 0.89 (*p* = 0.24, 95% CI, 0.84–0.92, *I*^*2*^ = 20.56%), 0.88 (*p*<0.001, 95% CI, 0.84–0.92, *I*^*2*^ = 82.75%), respectively. Sensitivity analysis showed no significant changes if any one of the studies was excluded. Publication bias was not detected in this meta-analysis.

**Conclusions:**

Our study suggests that SWE is a helpful method to appraise liver fibrosis severity. Future studies that validate these findings would be appropriate.

## Introduction

Liver fibrosis is a diffuse excessive deposition of extracellular matrix especially collagen material in the liver, which is a repair response mechanism after chronic liver injury of various causes [1]. Mild to moderate fibrosis is reversible while cirrhosis, the endstage outcome of fibrosis, is generally irreversible. Traditionally, although the liver biopsy remains as the gold standard to measure fibrosis as it offers precise diagnostic information, it could lead to various complications [2]. This could be affected by targeted sampling error, heterogeneity of liver fibrosis and limited sampling range. Some patients even can not accept the repeated sampling. Recently, emerging studies have depicted non-invasive ways to quantify the severity of liver fibrosis, such as serum markers, radiological imaging and elastography. Transient elastography (TE) is performed by using FibroScan, offering a quantifiable value of liver stiffness (kPa), however, the accuracy of TE evaluation of fibrosis severity is insufficient and the liver stiffness measurement (LSM) threshold of the different stages overlap. It is susceptible to abdominal effusion, obesity and breathing. Its performance in moderate liver fibrosis is especially low [3]. Acoustic radiation force impulse (ARFI) measures the average elasticity value in the region of interested (ROI) with the standard deviation, which can not provide elastic measurement in real time. Most imaging-based techniques do well at discerning patients at the extremes of fibrosis but could not exactly distinguish intermediate stages [4]. More recently, real-time shear wave elastography (SWE) that was first reported by Bercoff J et al in 2004, is a two-dimensional transient elastography technique based on the principle of Mach Cones for noninvasive evaluation of liver fibrosis [5, 6]. The key of the image is shear wave from radiation force generated by an amplitude modulated beam of focused ultrasound. These waves then are detected by a proper imaging modality [7]. The Young’s modulus is calculated via E = 3ρC^2^, where ρ is the density and the shear wave speed C is a time-of-flight estimation between two points during the shear wave propagation. The SWE mode shows a region of higher stiffness coded as a red area. Lesion margins are much better depicted on the elastography than on the ultrasound grayscale image [8]. Liver fibrosis also has a greater elastic coefficient than the normal hepatic tissue.

Despite its benefits, SWE also has some limitations. For example, SWE is difficult to be applied in skeletal muscle system due to the insufficient resolution, in which it has to depend on the legible two-dimensional images. There is a lack of large scale prospective studies on the application of SWE in the diagnosis of liver fibrosis and since there is limited number of studies, the effectiveness of this technique is still inconsistent [9–21]. Thereby, we conducted a meta-analysis to appraise the accuracy of SWE in them (**≥**F1; **≥**F2; **≥**F3 and F4).

## Material and Methods

### 2.1. Search strategy

This meta-analysis was performed on the basis of the PRISMA statement [22] (S1 and S2 Files). A systematic literature search was independently conducted by two individual investigators with the same method from PubMed, Embase, Web of science and Scopus up to May 13^th^, 2016, using the keywords “shear wave elastography”, “supersonic shear imaging”, “liver”, “hepar” and “hepatic” (S3 File). Data were obtained from the full-published paper and no language or race restriction was utilized. Furthermore, additional relevant published references were manually retrieved.

### 2.2. Selection criteria

The included studies had to meet the following criterias: (1) The study appraised the performance of SWE for the diagnosis of liver fibrosis. (2) Histopathological examination on a METAVIR fibrosis scale as the gold standard was applied to identify the classification of liver fibrosis. (3) Available data could be used to compute the true-positive, false-positive, true-negative and false-negative results of SWE for diagnosis of this disease. (4) Prospective and retrospective studies were included in this study. Researches with greater sample sizes were brought in when overlapping patient samples were recruited in more than one study.

### 2.3. Exclusion criteria

The exclusion criterias were as follows: (1) conference abstracts, case reports, review articles or inadequate data descriptions; (2) if the same study appeared in other publications, only studies with greater sample sizes were selected for this study.

### 2.4. Data extraction and quality assessment

According to the METAVIR scoring system, the histologic staging of fibrosis was classified into five stages: F0, no fibrosis; F1, early fibrosis (portal fibrosis without septa); F2, moderate fibrosis (portal fibrosis and few septa); F3, severe fibrosis (numerous septa without cirrhosis); and F4, cirrhosis [23]. Data were independently extracted by two investigators for these information, including the first author, date of publication, study design, population characteristics, country, male/female, age, BMI and cut-off value, with disagreements determined by consulting a third investigator. The quality assessment of diagnostic accuracy studies (QUADAS) questionnaire was applied to estimate the quality of the recruited articles, which was intended to estimate the internal and external validity of diagnostic accuracy studies included in the meta-analysis [24]. The QUADAS tool has 14 items appraising study design-related questions and the validity of the results in the study. Each item may be recorded as ‘‘yes”, ‘‘no” or ‘‘unclear”.

### 2.5. Statistical analysis

We computed the pooled sensitivity, specificity, positive and negative likelihood ratios, and the diagnostic odds ratio of SWE, and 95% CIs by a bivariate mixed-effects regression model. We can depict the sensitivity versus specificity and figure out the area under the curve (AUROC) through a summary receiver operating characteristic (SROC) curve [25]. We also estimated the level of between-study heterogeneity using the *I*^*2*^ test [26]. Additionally, publication bias was tested by regression of diagnostic odds ratio (lnDOR) against the inverse of the square root of the effective sample size (1/ESS^1/2^) and weighting by ESS. If *P* was less than 0.05 for the slope coefficient, it suggested marked asymmetry [27]. Threshold effects were evaluated by the Spearman correlation coefficient. Fagan nomograms were conducted as measures of post-test probabilities on the basis of the pooled sensitivity and specificity. All statistical analyses were performed by Stata 12.0 and MetaDiSc version 1.4 software.

## Results

### 3.1. Characteristics of eligible studies

**Fig 1:**
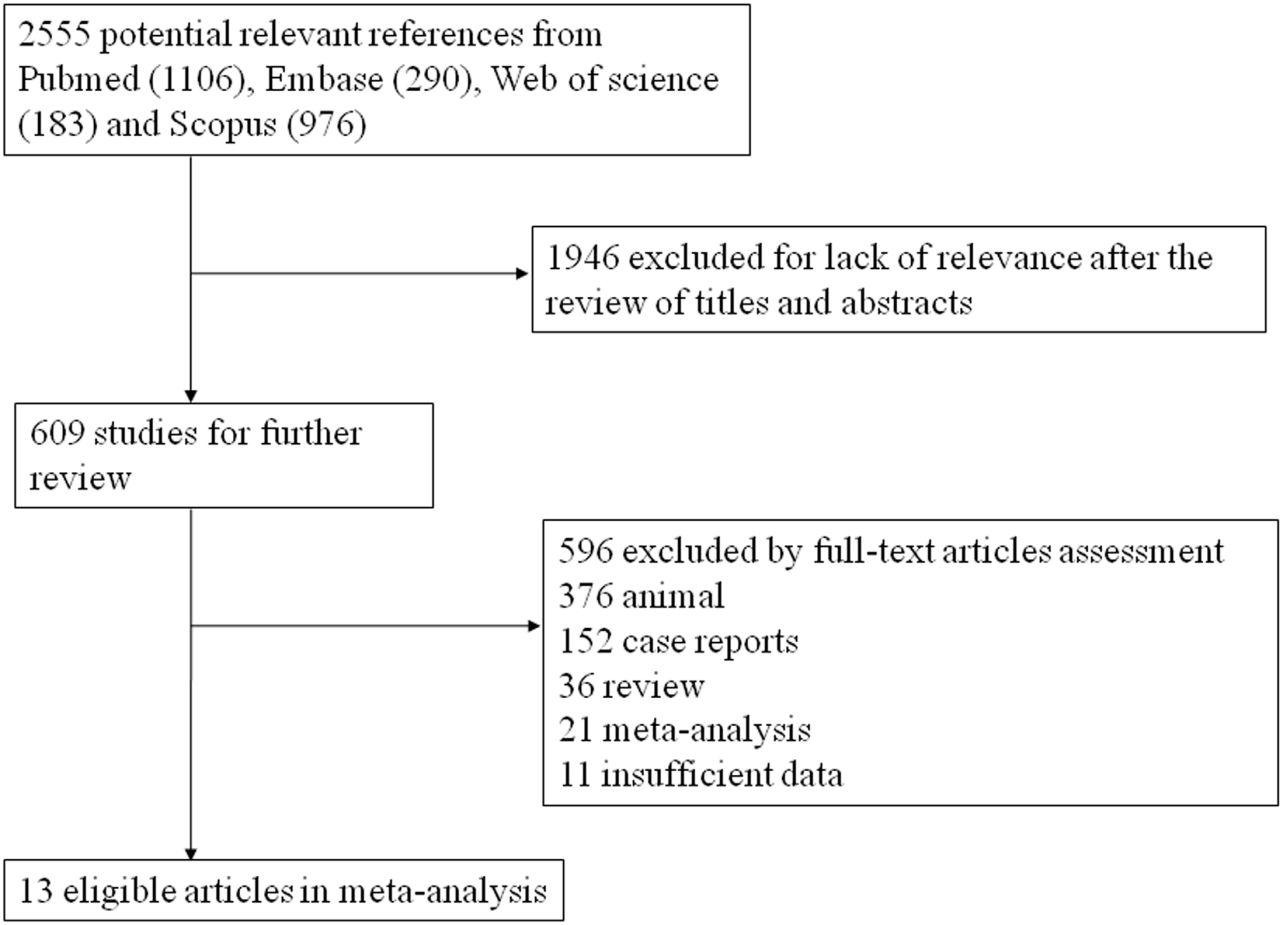
Flow diagram of the study selection process.

We identified 2303 cases (**≥**F1: 1226; **≥**F2: 2073; **≥**F3: 1836; F4: 1989) from 13 eligible studies up to May 13^th^, 2016 through the mentioned search strategies (Fig 1). The main characteristics of included studies for this meta-analysis are summarized in Table 1. They were all prospective cohort studies. The mean age of included patients was 36, and 57.1% were females. The most common underlying diseases were hepatitis B or C, and all patients underwent biopsy and the diagnosis was based on the histopathological examinations. The QUADAS scale showed that most of the studies were appraised as being of good quality (Table 2).

**Table 1:** Summaries of the studies included.

Author	Design	Population characteristics	Country	Male/female	Age (years)	BMI	Cut-off (Kpa)	Liver biopsy for fibrosis staging	Manufacturers of the instrument for SWE
Ferraioli G et al.2012	Prospective cohort study	Patients with chronic hepatitis C	Italy	87/34	44.8±11.9	25.4±3.8	≥F2:7.1;≥F3:8.7;F4:10.4	METAVIR	Aixplorer,SuperSonic Imagine,Aix-en-Provence,France
Leung VY et al.2013	Prospective cohort study	Patients with chronic hepatitis B	China	214/183	48.8±12.3	24.2±18.6	≥F1:6.5;≥F2:7.1;≥F3:7.9;F4:10.1	METAVIR	Aixplorer,SuperSonic Imagine,Aix-en-Provence,France
Cassinotto C et al.2014	Prospective cohort study	Patients with 8 hepatitis C;33 hepatitis B;145 non-alcoholic steatohepatitis;8 viral reactivation post-liver transplantation;5 sclerosing cholangitis;16 autoimmune diseases;7 hepatitis E;7 primary biliary cirrhosis;13 drug-related hepatitis;3 hemochromatosis;2 overlap syndrome;31 unexplained chronic cytolysis	France	188/161	54.8±14	27.4±6.4	≥F1:7.8;≥F2:8;≥F3:8.9;F4:10.7	METAVIR	Aixplorer,SuperSonic Imagine,Aix-en-Provence,France
Zeng J et al.2014	Prospective cohort study	Patients with chronic hepatitis B	China	a)169/37;b)82/22	a)36.3±9.4;b)37.2±10.9	a)22.3±3.2;b)22.1±3.4	≥F2:7.2;≥F3:9.1;F4:11.7	METAVIR	Aixplorer,SuperSonic Imagine,Aix-en-Provence,France
Beland M et al.2014	Prospective cohort study	Patients with 21 hepatitis C;15 elevated liver function tests;5 nonalcoholic steatohepatitis;3 cirrhosis;3 autoimmune hepatitis;2 hepatitis B;1 methotrexate therapy	USA	25/25	52	NA	≥F2:10.49 (1.87m/s), using the conversion formula from kPa to m/s as √(kPa/3)	METAVIR	Aixplorer,SuperSonic Imagine,Aix-en-Provence,France
Suh CH et al.2014	Retrospective cohort study	Patients with 123 nonsteatotic;73 hepatic steatosis	Korea	130/66	29.2±9.2	22.8±3.0	≥F1:6.2	METAVIR	Aixplorer,SuperSonic Imagine,Aix-en-Provence,France
Deffieux T et al.2015	Prospective cohort study	Patients with 44 hepatitis C;24 hepatitis B;11 healthy liver;11 non-alcoholic steatohepatitis;10 alcoholic liver;10 autoimmune diseases;2 hepatitis E;2 primary biliary cirrhosis;2 cryptogenic cirrhosis;2 steatosis;1 drug-related hepatitis;1 hepatocellular carcinoma	France	86/34	46.2±14.3	24.2±4.09	≥F2:8.9;≥F3:9.1;F4:10.2	METAVIR	Aixplorer,SuperSonic Imagine,Aix-en-Provence,France
Zheng J et al.2015	Prospective cohort study	Patients with 9 hepatitis C;164 hepatitis B;7 alcoholic liver;3 autoimmune diseases;4 primary biliary cirrhosis;4 drug-related hepatitis;7 unclassified	China	119/48	Male:36.6±9.7;female:39.7±11.8	21.6±3.4	NA	METAVIR	Aixplorer,SuperSonic Imagine,Aix-en-Provence,France
Tada T et al.2015	Retrospective cohort study	Patients with hepatitis C	Japan	23/32	61	21.3	≥F2:8.8	METAVIR	Aixplorer,SuperSonic Imagine,Aix-en-Provence,France
Samir AE et al.2015	Prospective cohort study	Patients with 43 hepatitis C;8 hepatitis B;1 alcoholic liver;18 autoimmune diseases;1 hemochromatosis;1 HIV and HCV coinfection;60 elevated liver function test;4 elevated liver function test after transplantation;	USA	70/66	49	NA	≥F2:7.29;≥F3:8.90;F4:9.59	METAVIR	Aixplorer,SuperSonic Imagine,Aix-en-Provence,France
Yoneda M et al.2015	Prospective cohort study	Patients with 117 hepatitis C;15 hepatitis B;7 alcoholic liver;13 non-alcoholic steatohepatitis;4 autoimmune diseases;6 primary biliary cirrhosis;9 primary sclerosing cholangitis;5 others	USA	115/59	57±12	30.1±4.1	a)≥F1:6.2;≥F2:7.9;≥F3:9.3;F4:11.4;b)≥F1:7.4;≥F2:8.6;≥F3:10.9;F4:14.7	METAVIR	Aixplorer,SuperSonic Imagine,Aix-en-Provence,France
Guibal A et al.2016	Prospective cohort study	Patients with 30 non-alcoholic steatohepatitis;22 hepatitis B or C;17 alcoholic liver;4 autoimmune hepatitis;4 chronic biliary disease;14 others	France	95/53	54.3±13.2	24.9±4.3	≥F2:8.8;≥F3:11.5;F4:18.1	METAVIR	Aixplorer,SuperSonic Imagine,Aix-en-Provence,France
Verlinden W et al.2016	Retrospective cohort study	Patients with 80 hepatitis C, including 26 coinfected with HIV	Belgium	63/17	43±10.2	NA	≥F2:8.5;≥F3:10.4;F4:11.3	METAVIR	Aixplorer,SuperSonic Imagine,Aix-en-Provence,France

NA:not available

**Table 2:** Quality assessment for included studies by QUADAS.

Author	1	2	3	4	5	6	7	8	9	10	11	12	13	14
	Representative spectrum of patients	Clear description of selection criteria	Adequate RS	Short time period between RS and index test	All patient verified by RS	Same RS used	RS independent of index test	Adequate index test description	Adequate RS	Blinding for index test	Blinding for RS	Clinical data available	Report of uninterruptible test result	Description of withdrawals
Ferraioli G et al. 2012	Y	Y	Y	Y	Y	Y	Y	Y	Y	Y	Y	Y	Y	NA
Leung VY et al. 2013	Y	Y	Y	Y	Y	Y	Y	Y	Y	Y	Y	Y	N	Y
Cassinotto C et al. 2014	Y	Y	Y	Y	Y	Y	Y	Y	Y	Y	Y	Y	N	Y
Zeng J et al. 2014	Y	Y	Y	Y	Y	Y	Y	Y	Y	Y	Y	Y	N	Y
Yoneda M et al. 2015	Y	Y	Y	Y	Y	Y	Y	Y	Y	NA	Y	Y	N	Y
Zheng J et al. 2015	Y	Y	Y	Y	Y	Y	Y	Y	Y	Y	Y	Y	Y	NA
Deffieux T et al. 2015	Y	Y	Y	Y	Y	Y	Y	Y	Y	NA	Y	Y	N	Y
Samir AE et al. 2015	Y	Y	Y	Y	Y	Y	Y	Y	Y	Y	Y	Y	N	NA
Beland M et al. 2014	Y	Y	Y	Y	Y	Y	Y	Y	Y	NA	NA	Y	N	NA
Suh CH et al. 2014	Y	Y	Y	Y	Y	Y	Y	Y	Y	NA	NA	Y	N	NA
Tada T et al. 2015	Y	Y	Y	Y	Y	Y	Y	Y	Y	Y	Y	Y	N	NA
Guibal A et al. 2016	Y	Y	Y	Y	Y	Y	Y	Y	Y	Y	Y	Y	N	NA
Verlinden W et al. 2016	Y	Y	Y	Y	Y	Y	Y	Y	Y	NA	NA	Y	N	NA

NA: not available

### 3.2. Diagnostic accuracy results

**Fig 2:**
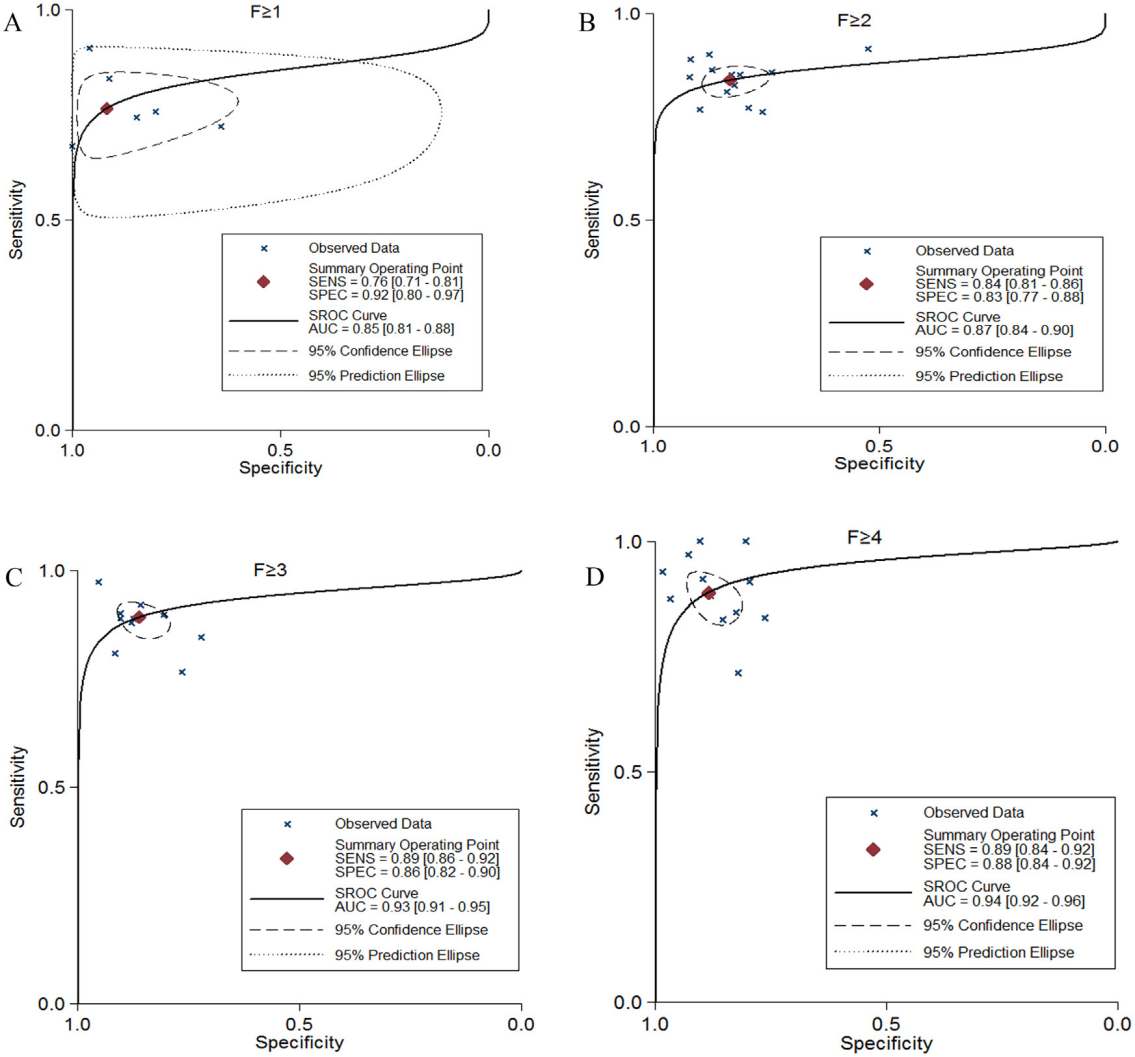
SROC curve of SWE in ≥F1-4 stagings (A, B, C and D) for liver fibrosis.

In the S1–S4 Figs, the pooled sensitivity, specificity, diagnostic odds ratio (DOR), positive, negative LRs and the area under the curve (AUC) of SWE for detecting accuracy of hepatic fibrosis severity were shown in Table 3. The summary area under the curve (AUC) was **≥**F1 0.85 (0.81–0.88), **≥**F2 0.87 (0.84–0.90), **≥**F3 0.93 (0.91–0.95) and F4 0.94 (0.92–0.96) (Fig 2). According to the Spearman correlation coefficient, threshold effect was not found significant in **≥**F1-4 stagings (**≥**F1–0.086, *p* = 0.872; **≥**F2–0.011, *p* = 0.972; **≥**F3–0.355, *p* = 0.284 and F4–0.406, *p* = 0.191). Fagan nomograms suggested that for all liver fibrosis severity, a positive test substantially increased the pre-test probability, while a negative test markedly reduced the pre-test probability (S4 Fig).

**Table 3:** Pooled sensitivity, specificity, positive likelihood ratio (+LR), negative likelihood ratio (-LR) and diagnostic oddsratio (DOR) (95% CI).

Pooled indexes	≥F1	≥F2	≥F3	≥F4
Sensitivity	0.76(0.71–0.81)	0.84(0.81–0.86)	0.89(0.86–0.92)	0.89(0.84–0.92)
*I*^*2*^	75.33	9.55	0	20.56
*P*	<0.001	0.35	0.56	0.24
Specificity	0.92(0.80–0.97)	0.83(0.77–0.88)	0.86(0.82–0.90)	0.88(0.84–0.92)
*I*^*2*^	79.36	86.56	75.73	82.75
*P*	<0.001	<0.001	<0.001	<0.001
Diagnostic OR	36.07(12.76–101.96)	25.16(17.40–36.38)	50.69(30.42–84.47)	60.89(31.26–118.61)
+LR	9.28(3.57–24.08)	4.92(3.65–6.61)	6.40(4.76–8.62)	7.68(5.30–11.13)
-LR	0.26(0.20–0.32)	0.20(0.17–0.23)	0.13(0.10–0.17)	0.13(0.09–0.18)
AUC	0.85(0.81–0.88)	0.87(0.84–0.90)	0.93(0.91–0.95)	0.94(0.92–0.96)

DOR: Diagnostic OR

+LR: Positive LR

-LR: Negative LR

In addition, according to the fibrosis etiology and due to small sample size on fatty liver disease, we have not performed a sensitivity test on it but do this test in 7 articles on hepatitis. These results of sensitivity and specificity were indicated as follows: ≥F2 0.84 (*p* = 0.13, 95% CI, 0.79–0.88, *I*^*2*^ = 40.78%), 0.88 (*p*<0.001, 95% CI, 0.82–0.92, *I*^*2*^ = 72.09%); ≥F3 0.90 (*p* = 0.22, 95% CI, 0.84–0.94, *I*^*2*^ = 32.37%), 0.91 (*p* = 0.37, 95% CI, 0.88–0.93, *I*^*2*^ = 3.91%); F4 0.90 (*p* = 0.1, 95% CI, 0.82–0.95, *I*^*2*^ = 49.27%), 0.90 (*p*<0.001, 95% CI, 0.84–0.94, *I*^*2*^ = 84.61%), respectively. The detailed results were indicated in S1 Table.

### 3.3. Sensitivity analysis and publication bias

Sensitivity analysis was performed to assess the stability of the results and found no significant change if any one of the studies was excluded. The publication bias were not detected in our meta-analysis (**≥**F1: t = -0.89, *p* = 0.425; **≥**F2: t = -1.18, *p* = 0.265; **≥**F3: t = -0.28, *p* = 0.782; F4: t = 0.44, *p* = 0.667).

## Discussion

Progressive fibrosis could result in serious consequences such as cirrhosis and hepatocellular carcinoma [28]. Assessment of the degree of liver fibrosis is vital for optimal therapeutic methods as well as the prognosis [29, 30]. Shear wave elastography, a new ultrasound-based elastographic method, is based on the traditional ultrasonography. It adds the data on tissue stiffness that may increase the accuracy of diagnosis. Depending on the present meta-analysis, we found that SWE may be an accurate technique in recognizing liver fibrosis.

This meta-analysis showed that the pooled sensitivity and specificity of SWE for liver fibrosis were satisfactory. The odds ratio (OR) is a common statistic in epidemiology, representing the strength of correlation between exposures and diseases. Diagnostic OR is defined as the probability of having a positive detection in patients with a true histological stage of the disease in contrast to patients without the disease. They regulate the negative and curvilinear relations between sensitivities and specificities, as well as consider heterogeneity between studies about the different thresholds [31]. This efficiently helps doctors in offering treatment to patients with early stage of the disease (DOR = 25–61 times). If hepatic fibrosis happens, SWE is a dominant diagnostic test to examine the severity due to its high sensitivity and specificity. Besides these, SROC curves for SWE in F1-4 severity indicated that the AUC values were approximately close to 1 (more than 0.85). Thus, SWE is considered a good test to assess the severity of liver fibrosis. Comparing with other stages, SWE has higher diagnostic OR in **≥**F4 staging, which means that it has better strengthen diagnosis of early-stage liver fibrosis. In comparison with previous meta-analysis by transient elastography [32] and ARFI [33], our study indicated that SWE has higher accuracy than TE and ARFI for assessing fibrosis severity. This resulted may because SWE recognized the diseased tissue hardness in real time. Threshold effect was not found significant in **≥**F1-4 stagings. Thus heterogeneity in other severity may be due to some factors such as study population, trial condition and disease severity. The positive LR of a diagnostic test detected how well the test can correctly find a disease severity. The higher the positive LR, the better the diagnostic test in accurately recognizing the true disease state. The negative LR of a diagnostic test could be utilized to find how well the test correctly eliminates a disease severity. The lower the negative LR, the better the diagnostic test in declining a disease severity. SWE has a high positive LR and a low negative LR for all severity which suggests that SWE may perform better in diagnosing the correct histological severity of liver fibrosis. SWE can also be used in patients with ascites or obesity, which is not affected by gas as SWE is based on the integration of a radiation force generated in tissue by an ultrasonic beam and an ultrafast imaging sequence acquired in real time with the propagation of the resulting shear waves [8].

In recent analysis, there may be some explanations for the accuracy of SWE in evaluating liver fibrosis severity. Lu YP *et al*. showed that SWE well recognized the change in liver stiffness and the progression of liver fibrosis in rabbit fatty liver models [34]. SWE allowed real-time test of coagulation necrosis generated by radiofrequency in pigs and this would be applied to observe US-guided thermal ablation [35]. Hepatic stellate cells mainly come from extracellular matrix proteins in hepatic fibrosis, as seen in type I collagen.

Our results should be explained in view of several limitations. First, the heterogeneity of the meta-analysis must be stated because the justification for pooling the data could be susceptible. In this analysis, heterogeneity may come from the variation in study population characteristics and the prevalence of liver fibrosis. Second, the accuracy of SWE mainly relies on the operator's performance. Various fibrosis patterns among diseases could lead to heterogeneous liver elasticity measurements. The mean liver stiffness value measured by SWE was not associated with the size of the region of interest (ROI), age or BMI, but it was affected by the different segments of the liver, the detection depth and gender [36]. Lastly, the overall sampling size of included studies was relatively small.

In spite of these limitations, this study offers considerable information that could inform physicians the accuracy of SWE. Thereby, SWE would be an inexpensive technique with widespread availability, particularly in areas with insufficient healthcare resources. In addition, we meticulously retrieved all published literature relevant to the research question and then extracted the data in duplicate through the described protocols to guarantee high quality and consistency in the final data. Missing data were searched from the authors and studies results were statistically merged to support these estimates of SWE for the screening of liver fibrosis severity.

## Conclusions

In conclusion, this meta-analysis indicated that SWE could be a promising tool to differentiate the severity of liver fibrosis. Future studies are also necessary to explore the potential confounding factors.

## Supporting Information

S1 Fig Forest plot displaying sensitivity (A) and specificity (B) of SWE to discern ≥F1 staging of liver fibrosis; Forest plot displaying sensitivity (C) and specificity (D) of SWE to discern ≥F2 staging of liver fibrosis. (TIF)

S2 Fig Forest plot displaying sensitivity (A) and specificity (B) of SWE to discern ≥F3 staging of liver fibrosis; Forest plot displaying sensitivity (C) and specificity (D) of SWE to discern F4 staging of liver fibrosis. (TIF)

S3 Fig DOR of SWE in ≥F1-4 stagings (A, B, C and D) for liver fibrosis. (TIF)

S4 Fig Fagan nomogram for ≥F1-4 stagings (A, B, C and D) for liver fibrosis using SWE. (TIF)

S1 File PRISMA Checklist. (DOC)

S2 File PRISMA 2009 Flow Diagram. (DOC)

S3 File Systematic literature search. (DOC)

S1 Table Pooled sensitivity, specificity, positive likelihood ratio (+LR), negative likelihood ratio (-LR) and diagnostic oddsratio (DOR) (95% CI) on hepatitis. (DOC)

## Abbreviations

SWE: shear wave elastography
TE: transient elastography
LSM: liver stiffness measurement
ARFI: acoustic radiation force impulse
ROI: region of interested
QUADAS: quality assessment of diagnostic accuracy studies
LR: likelihood ratio
AUROC: the area under the ROC curve
SROC: summary receiver operating characteristic
OR: odds ratio
AUC: area under the curve
