# Supplementary figures and images for: Diagnostic Accuracy of 2D-Shear Wave Elastography for Liver Fibrosis Severity: A Meta-Analysis
Source: PLoS One. 2016 Jun 14;11(6):e0157219. doi: 10.1371/journal.pone.0157219 (PMC4907490; doi:10.1371/journal.pone.0157219)

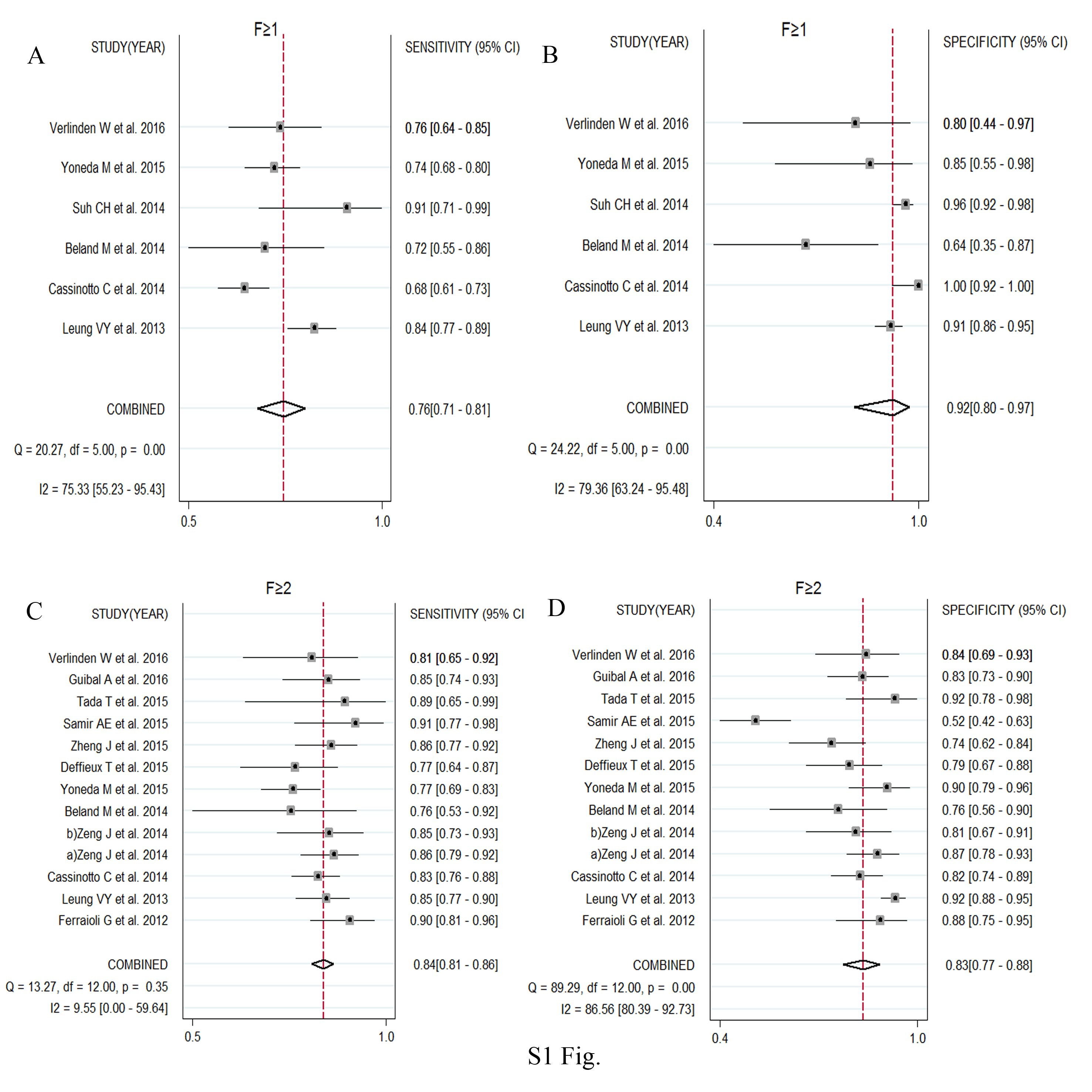

Supplement: S1 Fig — (TIF) [file pone.0157219.s001.tif]

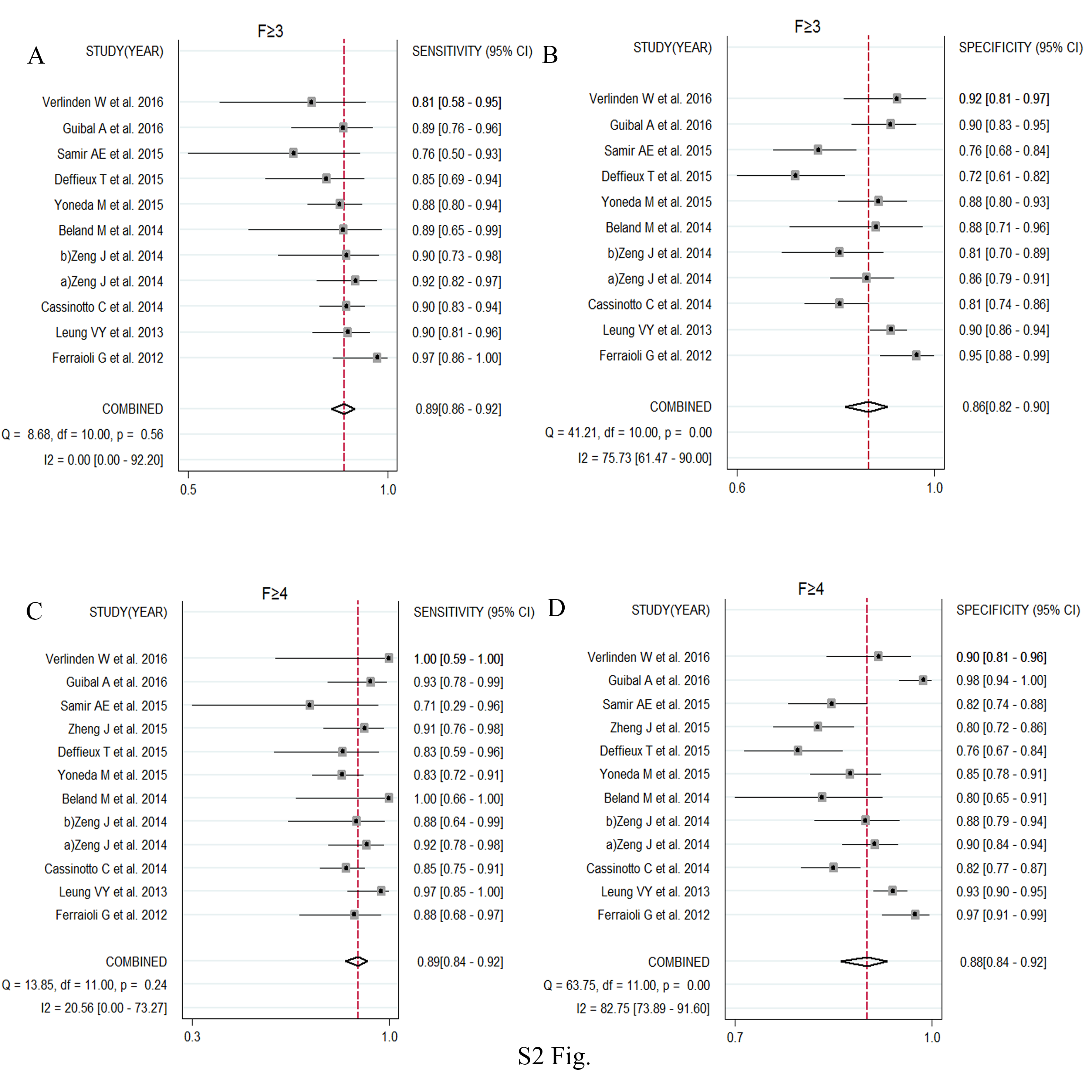

Supplement: S2 Fig — (TIF) [file pone.0157219.s002.tif]

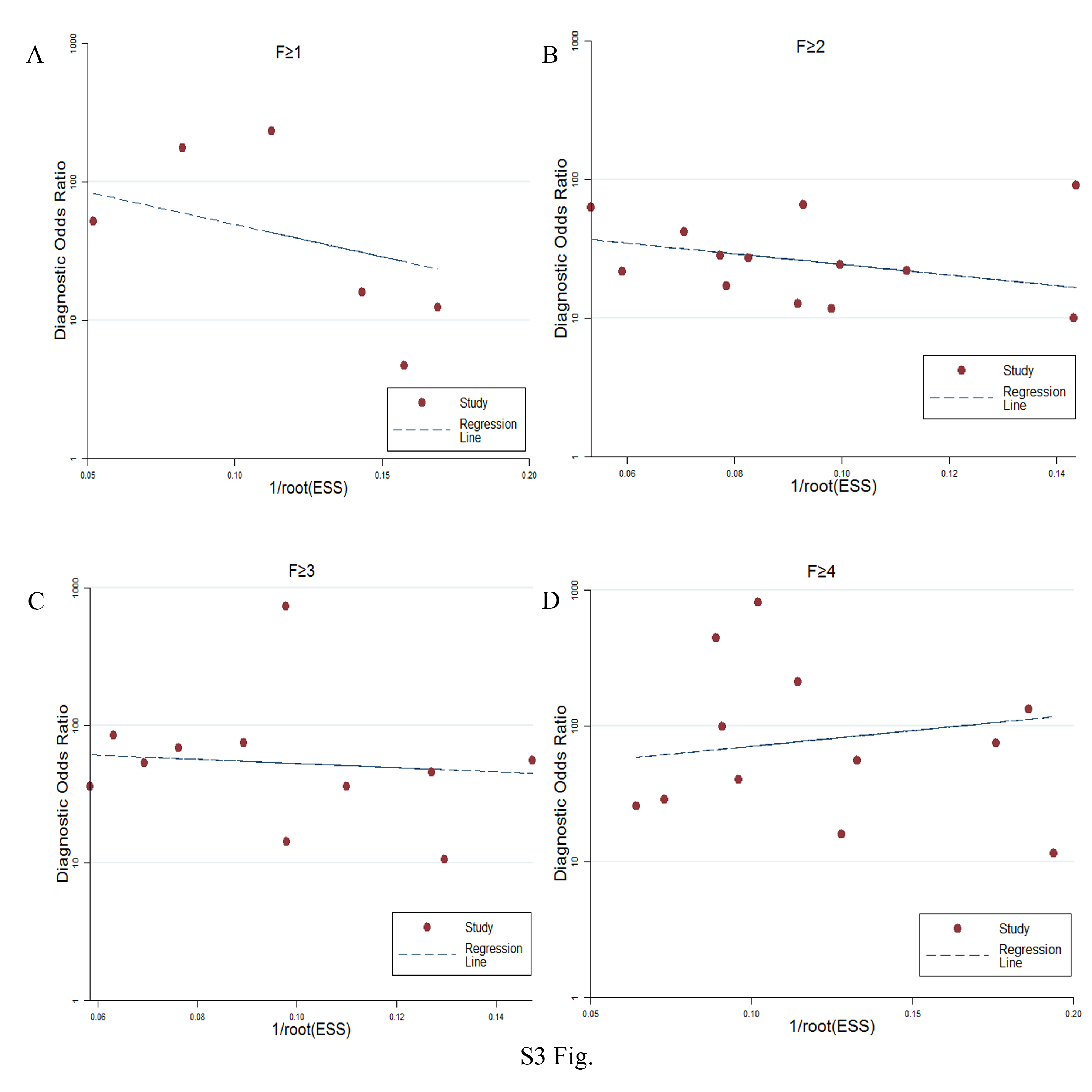

Supplement: S3 Fig — (TIF) [file pone.0157219.s003.tif]

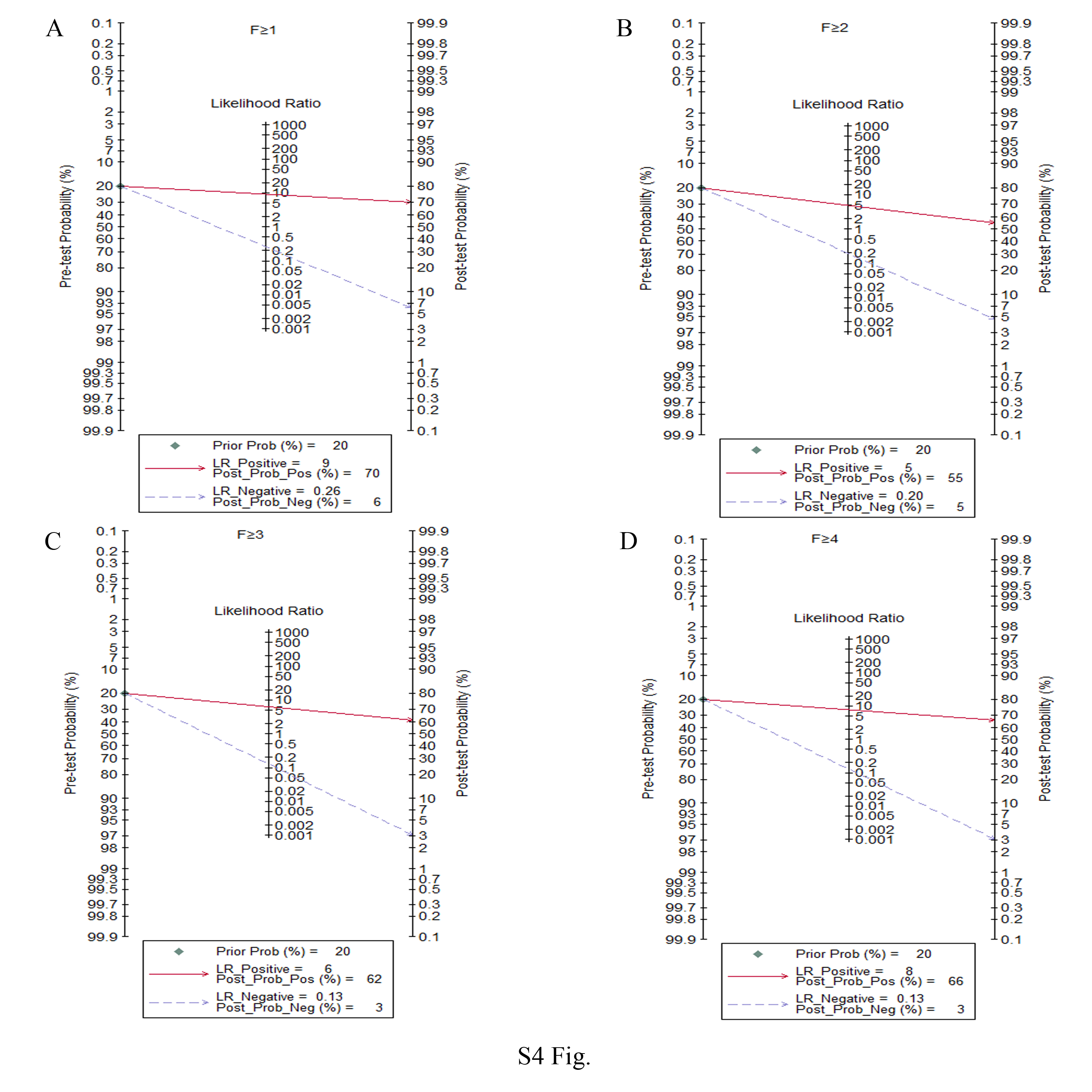

Supplement: S4 Fig — (TIF) [file pone.0157219.s004.tif]
