# Supplementary material for: Diagnostic Accuracy of 2D-Shear Wave Elastography for Liver Fibrosis Severity: A Meta-Analysis
Source: PLoS One. 2016 Jun 14;11(6):e0157219. doi: 10.1371/journal.pone.0157219 (PMC4907490; doi:10.1371/journal.pone.0157219)
Supplement: S2 File — (DOC) [file pone.0157219.s006.doc]

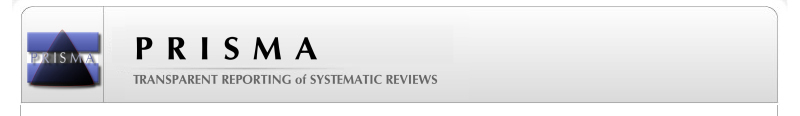
**PRISMA 2009 Flow Diagram**

**Screening**

**Included**

**Eligibility**

**Identification**

Records identified through database searching
(n =2555)

Additional records identified through other sources
(n = 6)

Records after duplicates removed
(n = 1506)

Records screened
(n = 882 )

Records excluded
(n =273)

Full-text articles assessed for eligibility
(n = 609)

Full-text articles excluded, with reasons
(n =596)

Studies included in qualitative synthesis
(n = 13)

Studies included in quantitative synthesis (meta-analysis)
(n = 13)
