# Supplementary material for: Diagnostic Accuracy of 2D-Shear Wave Elastography for Liver Fibrosis Severity: A Meta-Analysis
Source: PLoS One. 2016 Jun 14;11(6):e0157219. doi: 10.1371/journal.pone.0157219 (PMC4907490; doi:10.1371/journal.pone.0157219)
Supplement: S3 File — (DOC) [file pone.0157219.s007.doc]

**Search strategy:**

Pubmed (1950-present)

1. "shear wave elastography" OR "shear-wave elastography" OR SWE OR "supersonic shear imaging"
2. liver OR hepar OR hepatic
3. (liver stiffness) OR (liver fibrosis) OR cirrhosis
4. 1 AND 2 AND 3
5. " Elasticity Imaging Techniques"[Mesh]
6. " fibrosis "[Mesh]
7. "liver"[Mesh]
8. " liver cirrhosis"[Mesh]
9. 6 AND 7
10. 9 OR 8
11. 5 AND 9
12. 4 OR 11

Embase(1980-present)

1. ‘shear wave elastography’:ab,ti
2. ‘shear-wave elastography’:ab,ti
3. ‘SWE’:ab,ti
4. ‘supersonic shear imaging’:ab,ti
5. ‘liver’:ab,ti
6. ‘hepar’:ab,ti
7. ‘hepatic’:ab,ti
8. ‘liver stiffness’:ab,ti
9. ‘liver fibrosis’:ab,ti
10. ‘cirrhosis’:ab,ti
11. 1 OR 2 OR 3 OR 4
12. 5 OR 6 OR 7
13. 8 OR 9 OR 10
14. 11 AND 12 AND 13

Scoups

1. ALL ( “shear wave elastography” )
2. ALL ( "shear-wave elastography" )
3. ALL ( "SWE" )
4. ALL ( "supersonic shear imaging" )
5. 1 OR 2 OR 3 OR 4
6. ALL (liver)
7. ALL (hepar)
8. ALL (hepatic)
9. 6 OR 7 OR 8
10. ALL (liver stiffness)
11. ALL (liver fibrosis )
12. ALL (cirrhosis)
13. 10 OR 11 OR 12
14. 5 AND 9 AND 13

Web of science

1. TS=( Elasticity Imaging Techniques)
2. TS=(fibrosis)
3. TS=( liver)
4. TS=( liver cirrhosis)
5. 2 AND 3
6. 4 OR 5
7. 1 AND 6
8. TI=shear wave elastography
9. TI=shear-wave elastography
10. TI=SWE
11. TI=supersonic shear imaging
12. TI= liver
13. TI= hepar
14. TI= hepatic
15. TI=liver stiffness
16. TI=liver fibrosis
17. TI=cirrhosis
18. 8 OR 9 OR 10 OR 11
19. 12 OR 13 OR 14
20. 15 OR 16 OR 17
21. 18 AND 19 AND 20
22. 7 OR 21
