# Supplementary material for: Diagnostic Accuracy of 2D-Shear Wave Elastography for Liver Fibrosis Severity: A Meta-Analysis
Source: PLoS One. 2016 Jun 14;11(6):e0157219. doi: 10.1371/journal.pone.0157219 (PMC4907490; doi:10.1371/journal.pone.0157219)
Supplement: S1 Table — (DOC) [file pone.0157219.s008.doc]

**S1 Table** Pooled sensitivity, specificity, positive likelihood ratio (+LR), negative likelihood ratio (-LR) and diagnostic oddsratio (DOR) (95% CI) on hepatitis

| Pooled indexes | ≥F2 | ≥F3 | ≥F4 |
| --- | --- | --- | --- |
| Sensitivity | 0.84(0.79-0.88) | 0.90(0.84-0.94) | 0.90(0.82-0.95) |
| *I^2^* | 40.78 | 32.37 | 49.27 |
| *P* | 0.13 | 0.22 | 0.1 |
| Specificity | 0.88(0.82-0.92) | 0.91(0.88-0.93) | 0.90(0.84-0.94) |
| *I^2^* | 72.09 | 3.91 | 84.61 |
| *P* | <0.001 | 0.37 | <0.001 |
| Diagnostic OR | 36.20(21.68-60.45) | 86.93(43.98-171.85) | 82.91(29.83-230.46) |
| +LR | 6.71(4.50-10.01) | 9.79(7.17-13.38) | 9.03(5.32-15.31) |
| -LR | 0.19(0.14-0.24) | 0.11(0.07-0.18) | 0.11(0.06-0.21) |
| AUC | 0.91(0.88-0.93) | 0.96(0.94-0.97) | 0.95(0.93-0.97) |
